# Does Ecophysiology Determine Invasion Success? A Comparison between the Invasive Boatman *Trichocorixa verticalis verticalis* and the Native *Sigara lateralis* (Hemiptera, Corixidae) in South-West Spain

**DOI:** 10.1371/journal.pone.0063105

**Published:** 2013-05-17

**Authors:** Cristina Coccia, Piero Calosi, Luz Boyero, Andy J. Green, David T. Bilton

**Affiliations:** 1 Department of Wetland Ecology, Estación Biológica de Doñana-EBD (CSIC), Seville, Spain; 2 Marine Biology and Ecology Research Centre, University of Plymouth, Plymouth, United Kingdom; Estacion Experimental de Zonas Áridas (CSIC), Spain

## Abstract

**Background:**

*Trichocorixa verticalis verticalis*, a native of North America, is the only alien corixid identified in Europe. First detected in 1997 in southern Portugal, it has spread into south-west Spain including Doñana National Park. Its impact on native taxa in the same area is unclear, but it is the dominant species in several permanent, saline wetlands.

**Methodology/Principal Findings:**

We investigated whether the ecophysiology of this alien species favours its spread in the Iberian Peninsula and its relative success in saline areas. We compared physiological responses to heating (Critical Thermal maximum), cooling (Critical Thermal minimum) and freezing (Super Cooling Point) in the native *Sigara lateralis* and introduced *T. v. verticalis* acclimated to different temperatures and salinities. The larger *S. lateralis* generally outperformed *T. v. verticalis* and appeared to possess a broader thermal tolerance range. In both taxa, CT_max_ was highest in animals exposed to a combination of high conductivities and relatively low acclimation temperatures. However, CT_max_ was generally higher in *T. v. verticalis* and lower in *S. lateralis* when acclimated at higher temperatures. CT_min_ were lower (greater tolerance to cold) after acclimation to high conductivities in *T. v. verticalis*, and following acclimation to low conductivities in *S. lateralis*. Both acclimation temperature and conductivity influenced corixids' freezing tolerance; however, only in *T. v. verticalis* did SCP decrease after exposure to both high temperature and conductivity. *T. v. verticalis* showed a higher range of mean responses over all treatments.

**Conclusions:**

Whilst the native *S. lateralis* may have a broader thermal range, the alien species performs particularly well at higher salinities and temperatures and this ability may facilitate its invasion in Mediterranean areas. The greater plasticity of *T. v. verticalis* may further facilitate its spread in the future, as it may be more able to respond to climate shifts than the native species.

## Introduction

Freshwater habitats occupy less than 1% of the world's surface, but hold more than 7% of described species [1], with extensive local endemism [2], [3]. At the same time, however, inland water ecosystems and biological communities are affected by increasing numbers of alien species [4] and are amongst the most threatened in the world [1]. According to the DAISIE database, there are 296 invertebrate alien species in European inland waters [5]. However, the consequences of invasive invertebrate species for faunal composition, community structure and ecosystem functioning in freshwater systems are largely unknown, with the exception of a handful of taxa such as the red swamp crayfish *Procambarus clarkii* [6] and the zebra mussel *Dreissena polymorpha* [7].

Whilst some taxonomic groups (e.g. bivalves, crustaceans and gastropods) are well represented in alien invertebrate species lists, insects are highly under-represented, despite them dominating the world's freshwater fauna [8]. A recent addition to these lists is the water boatman *Trichocorixa verticalis verticalis* (Fieber, 1851) (Heteroptera, Corixidae), native to North America, but now occurring in temperate zones in other parts of the world such as South Africa, Iberia and Morocco [9]–[11]. In Europe, *T. v. verticalis* represents the only established alien waterbug [12]. In the Iberian Peninsula it was first recorded in 1997 in the Algarve in Portugal [13]. It is now successfully established and continues to spread, but is so far restricted to areas along the Atlantic coast [13] and in the Guadalquivir Estuary and surrounding parts of SW Spain [14], [15]. It is predicted to spread widely across Europe and the Mediterranean region in the future [16]

*T. v. verticalis* is now the dominant breeding corixid at several sites in and around Doñana National Park on the Guadalquivir Estuary [14], [15]. Part of its success appears to be related to its ability to live in hypersaline environments [17], and to colonize different kinds of habitats, including brackish and saline waterbodies [18]. This ability may enhance the competitive advantage of *T. v. verticalis* over other corixids in the face of global change. During the twentieth century, the wetlands in southern Spain and the rest of the Mediterranean region have become increasingly prone to development and extraction of fresh water [19], [20] and these factors, together with projected climate-induced changes in hydrology, increase salt concentrations in remaining waterbodies [21].

If native species are unable to respond to extreme conditions, either physiologically [22]–[25] or behaviourally [26], [27], they are likely to be excluded through interspecific competition with more tolerant species [28], [29]. Field data on the distribution of *T. v. verticalis* suggest that its physiological tolerance of salinity may be at least partly responsible for its competitive advantage over native corixids in the Doñana area [15]. Moreover, the effects of salinity and temperature on insect physiological tolerance can be synergistic or additive. Sánchez-Fernández *et al.* [30] for example, recently demonstrated how the interaction of these two environmental factors influences the thermal biology of adult *Nebrioporus* diving beetles, where cold tolerance increases following exposure to high salinities and low temperatures.

In this experimental study, we subjected *T. v. verticalis* and the native Palaearctic corixid *Sigara lateralis* (Leach, 1817) [31] to different combinations of temperature and salinity and compared several indicators of upper and lower thermal sensitivity of individuals of both species acclimated to different conditions. These two species are sympatric in southern Iberia, and frequently occur together in the same ponds, although *T. v. verticalis* is becoming the dominant corixid in some areas previously occupied by *S. lateralis* [14]. We specifically examined their critical thermal maximum (as a proxy for upper thermal limits), chill coma (as a proxy for lower thermal limits) [32], [33], and cold hardiness (supercooling point, often used as a measure of tolerance to low temperatures) [34], [35]. Differences in thermal tolerance and plasticity between native and invasive species can be used as predictors of their ability to persist, increase or decline in response to climate change. We explore whether exposure to different acclimation salinities and temperatures influence the thermal tolerance of the native and invasive species in an interactive manner, and examine the implications these have for the spread of *T. v. verticalis*.

## Materials and Methods

### Animal collection and maintenance

Adults of *Trichocorixa verticalis verticalis* and *Sigara lateralis* were collected during July and August 2010 using a D-framed pond net (500 µm mesh; 16×16 cm) from different sites in Doñana and the Odiel marshes (SW Spain). Permits for sampling in Doñana and Odiel were provided by the Consejería de Medio Ambiente, Junta de Andalucía. Conductivity of sampling sites ranged from 60 mS cm^−1^ (Odiel marshes) to 1.15 mS cm^−1^ (Doñana National Park) (See Table 1). Sites were chosen based on preliminary observations of corixid presence ([14], authors' unpublished data). After collection, corixids were transported to the laboratory inside plastic containers filled with damp aquatic vegetation and kept within thermally insulated polystyrene boxes in order to minimize thermal fluctuations and extremes as much as possible. In the laboratory, individuals were maintained in aquaria containing water close to the original conductivity, before being transferred to holding aquaria with water at conductivity 18 mS cm^−1^. When the original conductivity was >35 mS cm^−1^, to avoid acute exposure to experimental conditions, individuals were first maintained at 25–30 mS cm^−1^, before being transferred to 18 mS cm^−1^ (see Table 1). Aquaria were provided with sand and vegetation, and corixids were fed *ad libitum* with frozen chironomid larvae. Individuals were maintained on a natural photoperiod regime for 24 h before they were subjected to acclimation conditions, with a 12 h∶12 h D∶L regime.

**Table 1:** Collection sites in SW Spain, original conductivities and maintenance water conditions in the laboratory.

Sampling date	Arrival date	Sites	Original conductivity	Laboratory conductivity	Species
21/07/10	23/07/10	Veta la Palma (VLP)	11.59 mS cm^−1^	12 mS cm^−1^	*Tvv+Sl*
29/07/10	02/08/10	Odiel Marshes	60 mS cm^−1^	30 mS cm^−1^	*Tvv*
30/07/10	02/08/10	VLP-EBD	14 mS cm^−1^	14 mS cm^−1^	*Tvv*
30/07/10	02/08/10	FAO	2.8 mS cm^−1^	2.6 mS cm^−1^	*Tvv+Sl*
30/07/10	02/08/10	Caracoles	41 mS cm^−1^	25 mS cm^−1^	*Tvv+Sl*
19/08/10	20/08/10	FAO	1153 µS cm^−1^	1 mS cm^−1^	*Tvv+Sl*
19/08/10	20/08/10	VLP-EBD	36.5 mS cm^−1^	25 mS cm^−1^	*Tvv*
31/08/10	01/09/10	FAO	1.32 mS cm^−1^	1.32 mS cm^−1^	*Tvv+Sl*
31/08/10	01/09/10	Caño Guadiamar	6.76 mS cm^−1^	6 mS cm^−1^	*Tvv+Sl*

All sites are in Doñana except the Odiel Marshes (See [14], [15] for details]. VLP-EBD are individuals reared in mesocosms at the EBD (Estacíon Biólogica de Doñana-CSIC) but originating from Veta la Palma. *Tvv* = *Trichocorixa v. verticalis*; *Sl = Sigara lateralis*.

### Experimental setup and acclimation

Individuals were transferred to 3 L aquaria (with a maximum of 13 ind. of the same species in each aquarium) at 4 different conductivities: 1, 4, 12 and 18 mS cm^−1^, which corresponded to salinities of 0, 2.1, 6.8 and 10.6 ppt. Aquaria were kept for 72 h in either a climatic chamber set at 10 or 15°C or a water bath set at 25°C. Temperatures and salinities were chosen to simulate a range of conditions present at waterbodies where both species are found together [14], [36]. Whilst these conductivities do not span the entire range occupied by *T. v. verticalis* in the field (see above) they were chosen since preliminary experiments demonstrated that they were non-lethal in both taxa studied, allowing direct comparison of their responses to be conducted across a wide conductivity range. Waters of different conductivity were prepared by dissolving an appropriate quantity of salt (Instant Ocean, Aquarium Systems, Sarrebourg, France) in aerated artificial pond water, that consists of a solution of salts dissolved in double-distilled water, prepared according to a standardized protocol [37]. During the experiment we monitored water temperature and conductivity at 12 h intervals using a handheld multimeter (YSI 85, Yellow Springs, USA). Conductivity fluctuations, due to evaporation and/or differences in solubility, were corrected by dissolving small quantities of Instant Ocean or adding artificial pond water to aquaria. Aquaria were sealed with cling-film to reduce evaporation and to prevent individuals from escaping, whilst aeration was continuously provided. No food was provided 24 h prior to thermal tolerance limits being determined.

Following the exposure period, 10 individuals of each species were randomly removed from each treatment and further sub-divided into two equal-sized groups: one sub-group was used to measure critical thermal maximum (CT_max_) and the other to measure critical thermal minimum (CT_min_). The estimation of supercooling point (SCP) was undertaken in separate trials approx. 15 d after the determination of thermal limits, using the same procedure. After experiments, individuals were sexed using a stereo microscope and weighed to the nearest 0.001 g using a Sartorius 1204 MP2 balance (Sartorius Ltd, U.K.).

Thermal tolerance and supercooling point experiments were carried out in air given the impossibility to estimate freeze tolerance in water. This procedure provides an indication of the ability of a species to perform better than others at high or low temperatures in water as well as air [22]–[24], [30].

### Thermal tolerance experiments

Thermal tolerance tests commenced at the temperature at which individuals had been acclimated (see [38] for methodological details). A total of 240 individuals were used: 120 *S. lateralis* and 120 *T. v. verticalis.* Individuals were removed from their acclimation aquaria, quickly but carefully blotted on absorbent paper, and placed into a clean and dry well of a plastic multiwell culture plate. For CT_min_, specimens were placed individually into a generic 24-well plastic culture plate (Corning Ltd, Sunderland, UK), while for CT_max_ a modified plate was used with deeper wells to avoid escape during heating. In both cases, external bases were painted with white Tipp-Ex to allow easy visualization of temperature related responses. Plates were immersed in the water bath until only the upper edges (1–2 mm) were exposed, and affixed to the side of the bath with adhesive tape to prevent movements and thus water entering experimental wells. To further avoid escape, well plates were covered with a plastic lid between additions of individuals. Once the experiment started, lids were removed to allow full aeration and avoid the build-up of water vapour, which might have affected the thermal tolerance of individuals [39]. A maximum of 5 individuals were tested at any one time.

Thermal tolerance tests relied on a dynamic method, which involves increasing or decreasing test temperatures via a ramping program (±1°C min) until the end-point (see below) was observed. A rapid ramping rate was favoured as it allows observed responses to be related to the effect of different acclimations, and minimizes other undesired effects that may occur during slower ramping on thermal limits (see [40]). Experiments were performed with a Grant R5 water bath (12 l capacity) and a GP200 thermostatic controller (Grant Instruments Ltd., Cambridgeshire, England) connected to a computer. Grant Labwise software was used to construct and control temperature programs. The actual temperature within each well was measured directly using a calibrated digital thermometer (Omega_ HH11; Omega Engineering Inc., Stamford, CT, USA) equipped with an Omega® ‘precision fine wire thermocouple’ (type T – dia./ga. 0.08/0.13 Teflon). Distilled water and 70% ethylene glycol solutions were used as fluids inside the water bath to determine CT_max_ and CT_min_/SCP respectively.

CT_max_ and CT_min_ were defined using individual end-points represented by death (lethal point) at high temperatures, and chill coma (sub-lethal point) at low temperatures. Whereas death was readily identifiable in CT_max_ experiments (individuals never revived after cessation of movement), defining lower lethal limits was more difficult. At low temperatures, individuals exhibited total paralysis and were apparently dead (chill coma), but they would revive and recover full or partial locomotory abilities shortly after the end of the exposure period. As already documented for other insects [41], [24], both lethal limits and sublethal end-points (e.g. paralysis) provide an accurate picture of insect thermal biology. Consequently, we defined CT_min_ as the temperature at which individuals were paralysed, as the few corixids which recovered from the treatment were severely impaired in their locomotory ability and died shortly afterwards.

### Supercooling point experiment

The SCP is the temperature of spontaneous freezing at which a biological solution or a whole organism freezes when cooled below its equilibrium freezing temperature [42], [43]. During this experiment, the temperature at which individuals froze (SCP) was determined with a Campbell Scientific CR1000 datalogger equipped with an Omega ‘precision fine wire thermocouple’ (type T 1 mm long, 0.08 or 0.13 mm diameter) interfaced to a computer. Data were recorded and stored at 1 s intervals using Campbell Scientific PC400 software. Tests were carried out using a Grant R5 water bath (12 l capacity) and a GP200 thermostatic controller (Grant Instruments Ltd., Cambridgeshire, England) connected to a computer. Grant Labwise software was used to construct and control temperature programs.

A total of 115 individuals were tested: 60 *S. lateralis* and 55 *T. v. verticalis*. Individuals were removed from their exposure aquaria, quickly but carefully blotted on absorbent paper, and attached individually by the dorsum to an acetate disk with cyanoacrylic glue (Loctite, Henkel Ltd, Hempstead, UK). Individuals were introduced, one per well, into a 12-well plastic culture plate. A maximum of 5 animals were run concurrently in each experiment. The SCP was measured by supporting the thermocouple vertically on the insect's abdomen. Thermocouple movement was avoided by fixing individuals to the cell walls with BlueTack. Once ready, the individuals were transferred to the tank, and plates were covered with acetate lids to avoid thermal oscillations during the experiment. Individuals were cooled with a cooling ramp program (±1°C min^−1^), starting from the temperature at which individuals had been acclimated. The SCP of each individual was recorded as the lowest temperature reached before the start of the exothermic reaction caused by the latent heat of freezing of the animal's body fluids [44], [45]. Owing to a shortage of individuals, we were unable to test the SCP on individuals of *T. v. verticalis* exposed to 25°C and 18 mS cm^−1^.

### Statistical analyses

In order to assess the effect of exposure to different temperatures and conductivities on the thermal biology of *S. lateralis* and *T. v. verticalis*, we examined differences in CT_max_, CT_min_ and SCP with general linear models on untransformed data; with acclimation temperature (10, 15 or 25°C), acclimation conductivity (1, 4, 12 and 18 mS cm^−1^), and species (*T. v. verticalis* or *S. lateralis*) as fixed factors, and sex (male or female) as a random factor. With the exception of CT_max_, sex did not have a significant effect and was excluded from further analyses. Variances met assumptions for homoscedasticity (Levene's test, *P*>0.05), and data met the assumption of normality (Shapiro–Wilks test, *P*>0.05) for both CT_min_ and SCP as untransformed data, but not for CT_max_, even after log_10_ transformation. However, given our sample sizes, models employed were robust to deviations from normality [46], [47] and examination of residual plots for all data revealed satisfactory patterns. Model selection started by incorporating all predictors and the interactions between factors. Then, non-significant interactions were removed in a hierarchical, stepwise manner until a significant effect or interaction was found.

Body weight was not included in the overall model because it was not measured in all individuals of *T. v. verticalis*. We thus used a second model for only S. *lateralis* with the above factors together with body weight as a covariate. With the exception of CT_max_, body weight did not have a significant effect on *S. lateralis* thermal limits (*P*>0.05 for both CT_min_ and SCP), and was thus excluded from further analyses.

Finally, Bonferroni-corrected Estimate Marginal Means post-hoc tests were used for pairwise comparisons when any single factor or interaction was significant. All analyses were performed using SPSS version 17.0.

## Results

### Critical thermal maximum

**Figure 1:**
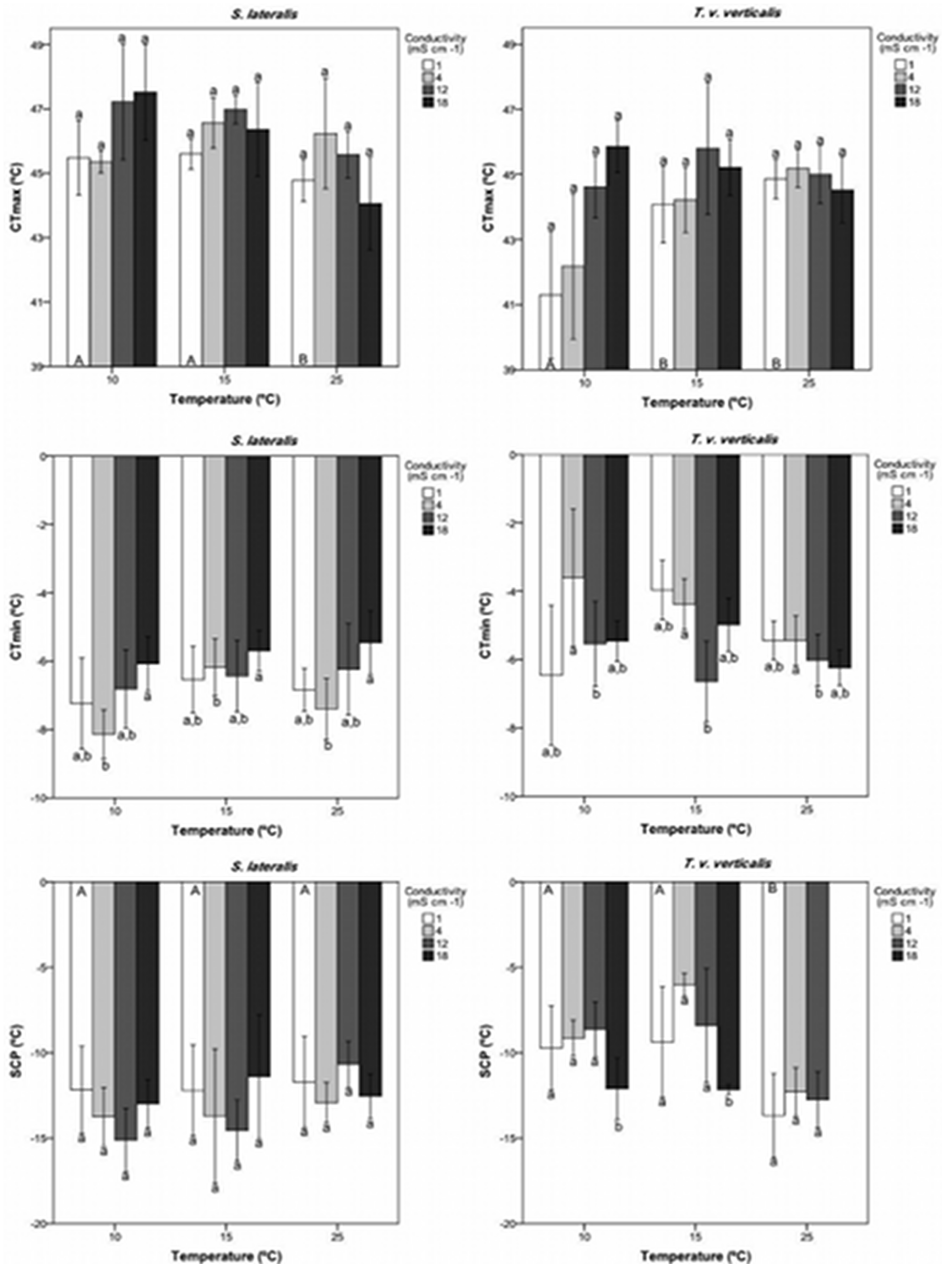
Thermal limits and freezing point of *T. v. verticalis* and *S. lateralis*. Histograms of mean ± SE critical thermal maximum (CT_max_), critical thermal minimum (CT_min_) and supercooling points (SCP) of *Sigara lateralis* and *Trichocorixa verticalis verticalis* acclimated to different temperatures (10, 15 and 25°C) and conductivities (1, 4, 12, 18 mS cm^−1^). Significantly different means within species (*P*<0.05) measured at different acclimation temperatures are indicated by different capital letters inside the histograms, whereas significantly different means measured at different conductivities within the same temperature treatment are indicated by different lower case letters above or below the histograms (according to Estimated Marginal Means tests with Bonferroni correction).

For both species, mean CT_max_ reached its maximum when individuals were acclimated at the lowest temperature (10°C) and the highest conductivity (18 mS cm^−1^) (Figure 1), whilst minimum CT_max_ were recorded at 10°C and 1 mS cm^−1^ for *T. v. verticalis* and 25°C and 18 mS cm^−1^ for *S. lateralis*.

In terms of their CT_max_, *S. lateralis* and *T. v. verticalis* responded differently to acclimation at different temperatures (temperature×species interaction *P*<0.001; Figure 1, Table 2 - Bonferroni tests maximum *P* = 0.035; Table S1). Mean CT_max_ was also significantly influenced by the interaction between temperature and conductivity (*P*<0.001; Figure S1, Table 2 - Bonferroni tests maximum *P* = 0.049; Table S1). Sex also had a strong influence (*P*<0.03; Table 2) on CT_max_ in both species' heat tolerance, CT_max_ being higher on average in females than males (Bonferroni tests maximum p = 0.030; Table S1).

**Table 2:** Effect of acclimation temperature (T), acclimation conductivity (C), species (Sp: *Trichocorixa verticalis verticalis* or *Sigara lateralis*) and sex on corixid critical thermal maximum (CT_max_) – General linear model.

Source	SS	d.f	MS	*F*	*P*
T	10.3	2	5.1	2.92	0.058
C	37.2	3	12.4	7.03	<0.001
Sp	51.8	1	51.8	29.37	<0.001
Sex	8.5	1	8.5	4.85	0.030
T×C	59.2	6	9.8	5.60	<0.001
T×Sp	32.7	2	16.3	9.28	<0.001
C×Sp	3.7	3	1.2	0.71	0.545

Sum of squares (SS); degrees of freedom (d.f.); mean square (MS), F-ratio (*F*), probability level (*P*).

Overall, CT_max_ was significantly higher in *S. lateralis* than in *T. v. verticalis* (Figure S2) at 10°C and 15°C (*P*<0.05) but not at 25°C (*P*>0.05). However, post-hoc comparisons showed that conductivity had a marginal influence on CT_max_ when animals were acclimated at higher temperatures. In contrast, CT_max_ was significantly lower for *S. lateralis* at 25°C than at other temperatures (Figure 1; Table S1).

*S. lateralis* was larger on average than *T. v. verticalis*, with mean (± SE) body weights of 5.35±1.28 mg and 3.46±0.73 mg, respectively. When *S. lateralis* was analysed separately with body weight as an additional covariate (Table 3), CT_max_ increased significantly with body weight (Pearson correlation *R* = 0.537, *P*<0.001) but sex no longer had a significant effect. Hence the effect of sex on CT_max_ seems to be a direct consequence of the lower body weight of males. Conductivity was the only other variable retaining a significant partial effect on CT_max_ once body weight was controlled for.

**Table 3:** Effects of acclimation temperature (T), acclimation conductivity (C) and weight (W) on the critical thermal maximum (CTmax) of *Sigara lateralis*– General linear model.

Source	SS	d.f	MS	*F*	*P*
T	6.4	2	3.2	2.43	0.098
C	17.1	3	5.7	4.34	0.009
W	18.2	1	18.2	13.88	0.001
T×C	11.7	6	1.9	1.40	0.206

Sum of squares (SS); degrees of freedom (d.f.); mean square (MS), F-ratio (*F*), probability level (*P*).

### Critical thermal minimum

Minimum CT_min_ were recorded at 10°C and 4 mS cm^−1^ for *S. lateralis* and 15°C and 12 mS cm^−1^ for *T. v. verticalis* (Figure 1). Maximum CT_min_ were recorded at 25°C and 18 mS cm^−1^ for *S. lateralis* and at 10°C and 4 mS cm^−1^ for *T. v. verticalis*. Mean lower thermal limit was significantly influenced by the interaction between species and conductivity (*P*<0.001; Table 4 - Bonferroni tests maximum *P* = 0.006; Table S2). Mean CT_min_ also differed significantly between species (*P*<0.001) with *S. lateralis* showing a higher tolerance to cold than *T. v. verticalis* (Bonferroni tests maximum *P*<0.001; Figure S2; Table S2). Acclimation temperature was not significantly related to mean CT_min_ in either species.

**Table 4:** Effect of acclimation temperature (T), acclimation conductivity (C) and species (Sp: *Trichocorixa verticalis verticalis* or *Sigara lateralis*) on corixid critical thermal minimum (CTmin) – General linear model.

Source	SS	d.f	MS	*F*	*P*
T	8.0	2	4.0	2.78	0.067
C	6.7	3	2.2	1.54	0.208
Sp	46.3	1	46.3	32.03	<0.001
T×C	13.8	6	2.3	1.59	0.158
T×Sp	6.1	2	3.0	2.11	0.126
C×Sp	31.5	3	10.5	7.27	<0.001

Sum of squares (SS); degrees of freedom (d.f.); mean square (MS), F-ratio (*F*), probability level (*P*).

CT_min_ was lower for *S. lateralis* (i.e., this species showed a greater tolerance to low temperatures) at both 1 and 4 mS cm^−1^. For *S. lateralis*, CT_min_ increased significantly as conductivity increased from 4 to 18 mS cm^−1^. In contrast, CT_min_ for *T. v. verticalis* decreased significantly as conductivity increased from 4 to 12 mS cm^−1^ (Table S2).

### Supercooling point

The minimum SCP for *S. lateralis* occurred when acclimated at 10°C and 12 mS cm^−1^, whilst the maximum for this species occurred when acclimated at 25°C and 12 mS cm^−1^ (Figure 1). For *T. v. verticalis*, minimum and maximum SCP occurred when acclimated at 25°C and 1 mS cm^−1^ and 15°C and 4 mS cm^−1^, respectively (Figure 1). Mean SCPs for *S. lateralis* and *T. v. verticalis* were influenced by acclimation at different temperatures (temperature×species interactions *P*<0.001; Table 5 - Bonferroni tests maximum *P* = 0.014; Table S3) and conductivities (conductivity×species interaction *P*<0.001; Table 5 - Bonferroni tests maximum = 0.026; Table S3). For both species, freezing point was significantly influenced by both conductivity (*P* = 0.041; Table 5; Bonferroni tests maximum = 0.044; Table S3) and acclimation temperature (*P* = 0.003; Table 5; Bonferroni tests maximum = 0.002; Table S3). Mean SCPs also differed strongly between species (*P* = 0.001; Table S3), being lower on average for *S. lateralis* (Bonferroni tests *P* = 0.001; Table S3).

**Table 5:** Effect of acclimation temperature (T), acclimation conductivity (C) and species (Sp: *Trichocorixa verticalis verticalis* or *Sigara lateralis*) on corixid supercooling point (SCP) – General linear model.

Source	SS	d.f	MS	*F*	*P*
T	76.0	2	38.0	6.29	0.003
C	51.9	3	17.3	2.86	0.041
Sp	77.3	1	77.3	12.81	0.001
T×C	32.8	6	5.4	0.90	0.495
T×Sp	190.6	2	95.3	15.78	<0.001
C×Sp	134.5	3	44.8	7.42	<0.001

Sum of squares (SS); degrees of freedom (d.f.); mean square (MS), F-ratio (*F*), probability level (*P*).

Overall, *S. lateralis* had a significantly lower SCP (i.e. greater tolerance to freezing) than *T. v. verticalis*. Such a significant effect was recorded at acclimation temperatures of 10 and 15°C, but was reversed at 25°C (Figure S2). At conductivities of 4 and 12 mS cm^−1^, *S. lateralis* had a significantly lower SCP than the alien corixid (Figure S2). For *T. v. verticalis* only, post-hoc tests showed that SCP varied significantly both with temperature and conductivity, decreasing as temperature increased to 25°C, and as conductivity increased to 18 mS cm^−1^ (Table S3).

## Discussion

*T. v. verticalis* and *S. lateralis* differed strongly in their physiological responses to heating, cooling and freezing; a finding in agreement with Chown *et al.* [48], who suggest that the form of physiological plasticity can be a key difference between invasive and native species. However, contrary to our expectations, *S. lateralis* generally outperformed *T. v. verticalis*, and appeared to possess a broader thermal tolerance range (*sensu* [24]). Both temperature and conductivity influenced corixid thermal tolerance. However, the effect of exposure to different temperatures and conductivities varied between upper and lower limits for the two species examined.

Although the temperatures recorded for CT_min_ and SCP are below those encountered by corixids under field conditions in our study area, their relative values and plasticities allow us to compare the relative ability of *Trichocorixa* and *Sigara* to cope with cold. The minimum air temperature recorded at the Palacio de Doñana in 2012 was −6°C and this matches the minimum value ever recorded in Doñana (February 1981; 2012), although temperatures below zero are not so unusual (http://www-rbd.ebd.csic.es/Seguimiento/mediofisico.htm). The CT_max_ values we recorded are ecologically very relevant, however, since the maximum air temperature often reaches 46°C in July–August. Corixids concentrate in shallow water whose temperature can exceed that of the air in summer. For example, in ponds frequented by the study species, water temperature reached 39°C in May 2007 (authors' unpublished data), whilst air temperature in the same month did not exceed 34°C.

### Critical thermal maximum

In terms of heat tolerance, the present study demonstrates that both species increase their CT_max_ in response to acclimation to a combination of high conductivity (18 mS cm^−1^) and low temperature (10°C). Such an effect was also recorded by Verween *et al.* [49], who found a trade-off between suboptimal temperature tolerance and high salinity in *Mytilopsis leucophaeata* (Mollusca, Bivalvia). Contrary to our initial expectations, acclimation to higher temperatures (25°C) did not improve heat tolerance in either corixid species. From our findings it appears that both species possess a similar heat shock response at the higher temperature employed.

Insects express heat shock proteins (HSPs) in response to both cold and osmotic shock [50], [51]. In *Drosophila*, exposure to low temperature results in heat shock protein upregulation when the animals are returned to higher temperatures [52], suggesting that the interaction between low temperature exposure and acute heating can also increase heat resistance [53]. Both processes may operate in the corixids in our study, suggesting that although HSP expression can vary among and within species [50], they appear to exhibit similar capacities to regulate HSP production under laboratory conditions. Such a plastic thermotolerance response has already been reported in many organisms [54] and here suggests that both corixids may use similar physiological mechanisms of acclimation when exposed to low temperatures and high salinity. On the other hand, the fact that both species did not elevate their heat tolerance after exposure to the higher temperature suggests that both species may maintain a high standing stock of HSPs in their cells. This mechanism often occurs in warm adapted organisms [54], and suggests that new warmer conditions experienced in SW Spain by *T. v. verticalis* compared to its native range may have led to some physiological changes as an adaptation to the local conditions.

From our data, *S. lateralis* appears to be generally more heat tolerant than *T. v. verticalis*. It is possible that the differences in maximum heat tolerance observed in the present study are at least partly based on differences in body size between the two species. Body size-mediated thermal acclimatory responses of upper thermal limit have previously been reported for diving beetles [30] and freshwater Crustacea [55], and could explain why the larger species *S. lateralis* showed a higher heat tolerance than *T. v. verticalis* here.

In general, warm adapted ectotherms possess great tolerance to heat [56], [57], but according to Stillman [56] they may have evolved this ability at the expense of their acclimatory capacity. Our results are in general agreement with Stillman's conclusion, since *S. lateralis* has a lower ability to acclimate CT_max_ in response to prior temperature exposure than *T. v. verticalis* (note how the alien shows greater magnitude of change in mean CT_max_ with temperature in Table S1), despite having the highest absolute CT_max_ overall. The fact that *Trichocorixa* apparently has greater plasticity to heat than *S. lateralis* may make it better able to respond to sudden temperature shifts in nature, something which may favour its spread.

### Critical thermal minimum

Whilst the native *S. lateralis* generally entered chill coma at lower temperatures, the response to acclimation conductivity was species specific. Whereas *S. lateralis* increased CT_min_ at lower conductivities, the opposite occurred for *T. v. verticalis*. Several previous studies have found effects of salinity on cold tolerance in other ectotherms, including *Nebrioporus* diving beetles, and fishes including the blackchin tilapia (*Sarotherodon melanotheron*) and the red drum (*Sciaenops ocellatus*) ([30], [58], but see [59]). Doñana and surrounding areas such as the Odiel marshes are characterized by a Mediterranean subhumid climate with rainfall between late September and early April, hot and dry summers, and mild winters [60]. Salinity varies spatially and temporally, but many ponds and marshes in Doñana are oligohaline during the winter [60]. Given that *S. lateralis* overwinters as adults, our results suggest that its ability to better remain active at lower conductivities may reflect the ability to minimize energetic costs for osmoregulation during the winter season. However, such an adaptation for winter survival could bring a high cost for *S. lateralis* in terms of development, fecundity and longevity [50].

Cold hardiness and desiccation resistance are mechanistically linked, and one is thought to originally have developed from the other [61]. Amongst *Drosophila* species, widespread species possess higher levels of resistance to both desiccation and cold [62]. Furthermore, this lack of genetic limitation in resistance traits appears to help drive *Drosophila* distribution patterns. Thus, it is plausible that *T. v. verticalis* possesses such desiccation-inducible genes that are also induced by the desiccating effect of increases in ambient salinity. In response to osmotic stress at higher conductivities, these genes produce solutes that enhance cold tolerance [63]. In its native habitats, *T. v. verticalis* is considered to be a euryhaline insect [64] and often occurs in brackish and saline waters [14]. As with *S. lateralis*, *T. v. verticalis* overwinters as adults, but contrary to the native species, seems well adapted to overwinter in higher salinity waterbodies, like estuarine fish ponds [14], [36]. In this context, our results suggest that the osmoregulatory ability of *T. v. verticalis* may allow this alien to spend the cold season in saline wetlands, where it probably also achieves continuous reproduction and development. This would help explain its successful colonization of Doñana, especially its dominance in permanent, saline fish ponds [14], [15].

We detected no effect of temperature of acclimation on CT_min_, contrary to many previous studies on insects (e.g. [65], [66], [30]). This absence of acclimatory ability shows limited temperature-dependent phenotypic plasticity for CT_min_ in our study species. Freezing winter temperatures are unusual in wetlands of southern Iberia, and these populations may not need well developed acclimatory abilities, which are known to have costs related to the severity of the stress [67]. In contrast, much colder winter temperatures are observed in the native range of *T. v. verticalis* along the east coast of North America (www.worldclim.org), and it would be interesting to compare native and invasive populations in this regard.

### Supercooling point

Both corixid species are freeze-avoiding insects, as they both show pre-freeze mortality and the SCP represents their lower lethal limit to survival. Moreover, a decrease in SCP is likely to be part of their seasonal cold-hardening strategy [68]. Different factors contribute to the enhancement of SCP capacity in insects, especially body size [69]. However, we didn't find an effect of intraspecific size variation in our study.

In the case of *T. v. verticalis*, cold hardiness was higher after acclimation to both higher temperatures and conductivities. This may result from physiological adjustments that probably involve heat protectant accumulation in response to high temperature and water loss regulation in response to osmoregulatory stress. As temperature increases, *T. v. verticalis* increase its heat tolerance, perhaps by HSP upregulation. The ability of HSPs to improve both heat and cold stress has been well documented in *Drosophila* species ([53], for reviews see [50]), as has the influence of dehydration on insects' cold hardiness [42].

Since we did not observe any influence of either acclimation temperature or salinity on SCP in *S. lateralis*, it is possible that the native and exotic species differ fundamentally in their physiological ability to supercool. This lack of acclimatory ability of SCP in *S. lateralis* suggests that *T. v. verticalis* may in fact be better able to survive temperature and salinity fluctuations, despite the fact that it generally exhibited higher CT_min_ and SCPs than *S. lateralis*.

### Implications for the invasion of *T. v. verticalis*

Overall, we found the native *S. lateralis* to be more thermally tolerant than the invasive *T. v. verticalis*, and our results may explain why *S. lateralis* remains dominant in freshwater ponds in the Doñana area, where *T. v. verticalis* is rare and has not been confirmed as a breeding species [14]. However, our study supports the hypothesis that an ability to cope with environmental fluctuations, and a high resistance to salinity, favours the invasion of *T. v. verticalis* in the Mediterranean region. The tolerance of *T. v. verticalis* to both heat and freezing increases following exposure to high conductivities. The mean salinity of remaining natural wetlands in the Mediterranean basin is much higher than in northern Europe [70], [21], partly because freshwater wetlands have been drained more extensively [19]. Under a scenario of further climatic warming, greater evapotranspiration rates are likely to promote further increases in salinity [21], and as a consequence, species able to cope with higher salinities may benefit from ongoing global change. The ability of *T. v. verticalis* to survive and reproduce in waters of relatively high conductivity during winter may be central to its success. The regular droughts occurring in the Mediterranean region mean that some winters see so little rain that many freshwater marshes do not flood, and in regions such as Doñana, this leaves water only in brackish fish ponds or coastal salt-pans which are now dominated by *T. v. verticalis* [71]. Our results suggest that *T. v. verticalis* has higher cold tolerance than *S. lateralis* in such habitats, a factor which is likely to contribute to its overwinter survival and reproduction. Saline waters may act as sources of the invasive *T. v. verticalis* for the surrounding freshwater habitats in Doñana and elsewhere, and its broad salinity tolerance and ongoing salinization of aquatic habitats may play important roles during the invasion.

Plasticity is a recognized characteristic of good invaders [72], [73] and the thermal physiology of *T. v. verticalis* is consistent with this pattern. The greater range of mean responses recorded across our 12 experimental treatments in *T. v. verticalis* compared to *S. lateralis* (4.56 vs 3.46°C for CT_max_; 3.04 vs 2.68°C for CT_min_; 7.65 vs 3.89°C for SCP) all point to greater plasticity in the invader. In addition to its physiological abilities, life history characteristics may play a central role in the invasion success of *T. v. verticalis*. According to Sol *et al.* [74], successful invaders can face the ecological pressure posed by the newly invaded environment by allocating reproductive efforts over several breeding events. *T. v. verticalis* has multiple generations a year in permanent fish ponds in Doñana (authors unpublished data), whereas *S. lateralis* is bivoltine [31]. Whilst there are limited data on the life-history of native populations of *T. v. verticalis* in the Americas, it appears that the warmer climate of the Mediterranean area may have allowed this species to switch to reproducing throughout the year, as suggested in previous studies [15]. Such responses can occur rapidly following invasion. Japanese populations of the fall webworm (*Hyphantria cunea*, Lepidoptera) have shifted from being bivoltine to trivoltine in 25 years when exposed to new environmental conditions [75]. In *T. v. verticalis*, the ability to reproduce throughout the year, together with an apparently greater plasticity to heat, cold and salinity could facilitate its survival in the face of new environmental conditions, and indeed facilitate its spread as climate change proceeds.

Finally, whilst *T. v. verticalis* occurs in sympatry with the native *S. lateralis* in Spain [14], it also appears to overlap the salinity niche of some halophilic European corixids such as *S. selecta* (Fieber, 1848) and *S. stagnalis* (Leach, 1817) [36]. Future research should address possible interactions with these other species, since the outcomes of these encounters may not be identical

## Supporting Information

Figure S1 **Interactive effect of temperature and conductivity on mean CTmax.** Histograms are mean ± SE critical thermal maximum (CT_max_) of *Sigara lateralis* and *Trichocorixa verticalis verticalis* acclimated to different temperatures (10, 15 and 25°C) and conductivities (1, 4, 12, 18 mS cm^−1^). Significantly different means (*P*<0.05) between different acclimation temperatures measured at the same acclimation conductivity are indicated by different capital letters inside the histograms, whereas significantly different means measured at different conductivities at the same acclimation temperature are indicated by different lower case letters above or below the histograms (according to Estimated Marginal Mean test with Bonferroni correction).
(TIF)

Figure S2 **Thermal limit and freezing point differences between** ***T. v. verticalis*** **and** ***S. lateralis*** **.** Histograms of mean ± SE critical thermal maximum (CT_max_), critical thermal minimum (CT_min_) and supercooling points (SCP) of *Sigara lateralis* and *Trichocorixa verticalis verticalis* acclimated to different temperatures (10, 15 and 25°C) and conductivities (1, 4, 12, 18 mS cm^−1^), according to linear model output. Significantly different means between species (*P*<0.05) measured at different acclimation temperatures are indicated by different capital letters inside the histograms, whereas significantly different means between species measured at different conductivities are indicated by different lower case letters above or below the histograms (according to Estimated Marginal Mean test with Bonferroni correction).
(TIF)

Table S1 **Significantly different mean CT_max_ (Estimated Marginal Means tests with Bonferroni correction) from** **Table 2** **according to acclimation temperature (T), acclimation conductivity (C), species (Sp:** ***Trichocorixa verticalis verticalis*** **or** ***Sigara lateralis*** **) and sex (1 = male; 2 = female).** These tests refer to partial effects from the final model.
(DOCX)

Table S2 **Significantly different mean CT_min_ (Estimated Marginal Means tests with Bonferroni correction) from** **Table 4** **according to acclimation conductivity (C) and species (Sp:** ***Trichocorixa verticalis verticalis*** **or** ***Sigara lateralis*** **).** These tests refer to partial effects from the final model.
(DOCX)

Table S3 **Significantly different mean SCPs (Estimate Marginal Means tests with Bonferroni correction) from** **Table 5** **according to acclimation temperature (T), acclimation conductivity (C) and species (Sp:** ***Trichocorixa verticalis verticalis*** **or** ***Sigara lateralis*** **).** These tests refer to partial effects from the final model.
(DOCX)
