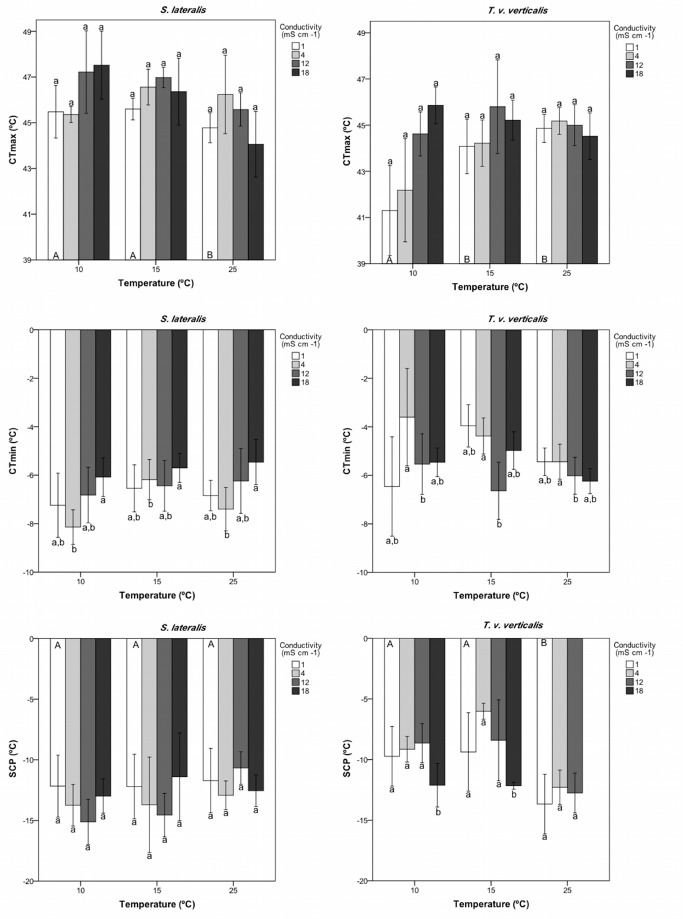# Correction: Does Ecophysiology Determine Invasion Success? A Comparison between the Invasive Boatman *Trichocorixa verticalis verticalis* and the Native *Sigara lateralis* (Hemiptera, Corixidae) in South-West Spain

**DOI:** 10.1371/annotation/2c817fcd-acf8-49ea-82cb-74776a3eeb9b

**Published:** 2013-11-07

**Authors:** Cristina Coccia, Piero Calosi, Luz Boyero, Andy J. Green, David T. Bilton

Figure 1 has been updated for better readability. Please see correct Figure 1 here: 

**Figure:**