# Supplementary figures and images for: Does Ecophysiology Determine Invasion Success? A Comparison between the Invasive Boatman Trichocorixa verticalis verticalis and the Native Sigara lateralis (Hemiptera, Corixidae) in South-West Spain
Source: PLoS One. 2013 May 17;8(5):e63105. doi: 10.1371/journal.pone.0063105 (PMC3656867; doi:10.1371/journal.pone.0063105)

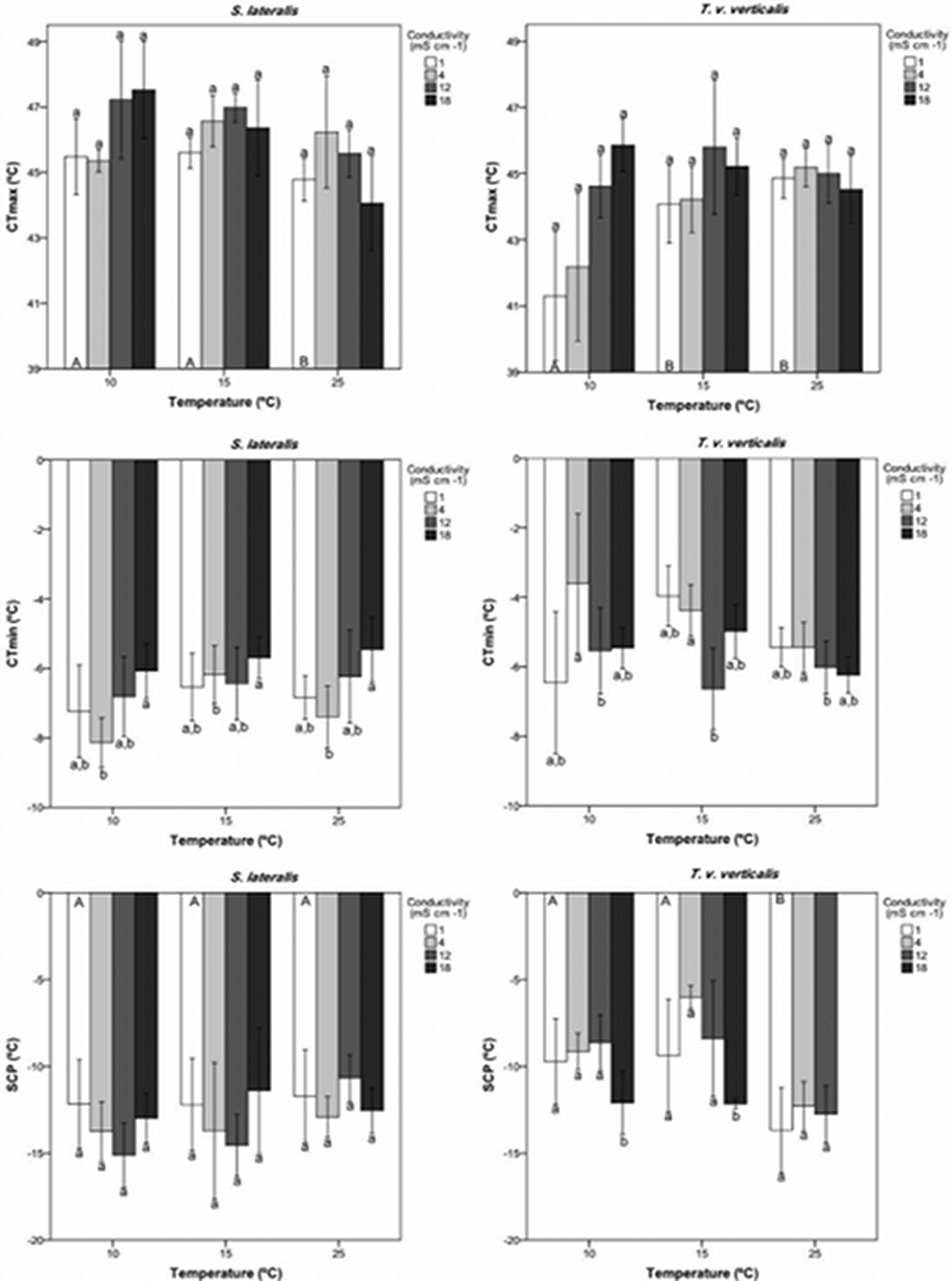

Supplement: Figure S1 — Interactive effect of temperature and conductivity on mean CTmax. Histograms are mean ± SE critical thermal maximum (CTmax) of Sigara lateralis and Trichocorixa verticalis verticalis acclimated to different temperatures (10, 15 and 25°C) and conductivities (1, 4, 12, 18 mS cm−1). Significantly different means (P<0.05) between different acclimation temperatures measured at the same acclimation conductivity are indicated by different capital letters inside the histograms, whereas significantly different means measured at different conductivities at the same acclimation temperature are indicated by different lower case letters above or below the histograms (according to Estimated Marginal Mean test with Bonferroni correction). (TIF) [file pone.0063105.s001.tif]

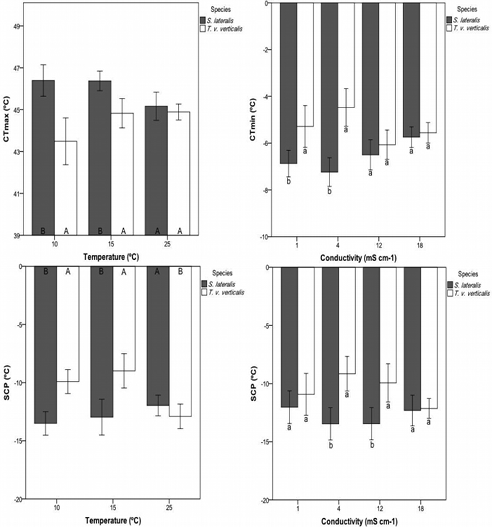

Supplement: Figure S2 — Thermal limit and freezing point differences between T. v. verticalis and S. lateralis . Histograms of mean ± SE critical thermal maximum (CTmax), critical thermal minimum (CTmin) and supercooling points (SCP) of Sigara lateralis and Trichocorixa verticalis verticalis acclimated to different temperatures (10, 15 and 25°C) and conductivities (1, 4, 12, 18 mS cm−1), according to linear model output. Significantly different means between species (P<0.05) measured at different acclimation temperatures are indicated by different capital letters inside the histograms, whereas significantly different means between species measured at different conductivities are indicated by different lower case letters above or below the histograms (according to Estimated Marginal Mean test with Bonferroni correction). (TIF) [file pone.0063105.s002.tif]
