# Supplementary material for: Does Ecophysiology Determine Invasion Success? A Comparison between the Invasive Boatman Trichocorixa verticalis verticalis and the Native Sigara lateralis (Hemiptera, Corixidae) in South-West Spain
Source: PLoS One. 2013 May 17;8(5):e63105. doi: 10.1371/journal.pone.0063105 (PMC3656867; doi:10.1371/journal.pone.0063105)
Supplement: Table S1 — Significantly different mean CTmax (Estimated Marginal Means tests with Bonferroni correction) from Table 2 according to acclimation temperature (T), acclimation conductivity (C), species (Sp: Trichocorixa verticalis verticalis or Sigara lateralis ) and sex (1 = male; 2 = female). These tests refer to partial effects from the final model. (DOCX) [file pone.0063105.s003.docx]

**Table S1**.

| **conductivity vs temperature** | | | | | |
| --- | --- | --- | --- | --- | --- |
| C | (I)T | (J)T | Mean Difference (I-J) | Std. Error | Sig. |
| 1 | 10 | 15 | -1.45 | 0.59 | 0.049 |
|  |  | 25 | -1.49 | 0.59 | 0.040 |
| 4 | 10 | 15 | -1.68 | 0.59 | 0.016 |
|  |  | 25 | -2.00 | 0.59 | 0.003 |
| 18 | 10 | 25 | 2.26 | 0.59 | 0.001 |
| **temperature vs conductivity** | | | | | |
| T | (I)C | (J)C | Mean Difference (I-J) | Std. Error | Sig. |
| 10 | 1 | 12 | -2.39 | 0.59 | 0.001 |
|  |  | 18 | -3.23 | 0.59 | <0.001 |
|  | 4 | 12 | -2.08 | 0.59 | 0.004 |
|  |  | 18 | -2.92 | 0.59 | <0.001 |
| **temperature vs species** | | | | | |
| T | (I)Sp | (J)Sp | Mean Difference (I-J) | Std. Error | Sig. |
| 10 | *Sl* | *Tvv* | 2.69 | 0.43 | <0.001 |
| 15 | *Sl* | *Tvv* | 1.33 | 0.43 | 0.002 |
| **species vs temperature** | | | | | |
| Sp | (I)T | (J)T | Mean Difference (I-J) | Std. Error | Sig. |
| *Sl* | 10 | 25 | 1.09 | 0.42 | 0.035 |
|  | 15 | 25 | 1.10 | 0.42 | 0.031 |
| *Tvv* | 10 | 15 | -1.37 | 0.42 | 0.004 |
|  |  | 25 | -1.46 | 0.42 | 0.002 |

| **Conductivity** | | | | | |
| --- | --- | --- | --- | --- | --- |
| (I)C | (J)C | Mean Difference (I-J) | Std. Error | Sig. |  |
| 12 | 1 | 1.44 | 0.34 | <0.001 |  |
| 18 | 1 | 1.17 | 0.34 | 0.006 |  |
| **species** | | | | | |
| (I)Sp | (J)Sp | Mean Difference (I-J) | Std. Error | Sig. |  |
| *Sl* | *Tvv* | 1.39 | 0.25 | <0.001 |  |
| **sex** | | | | | |
| (I)Sex M | (J)Sex F | Mean Difference (I-J) | Std. Error | Sig. |  |
| 1 | 2 | -0.69 | 0.31 | 0.030 |  |
