# Supplementary material for: Does Ecophysiology Determine Invasion Success? A Comparison between the Invasive Boatman Trichocorixa verticalis verticalis and the Native Sigara lateralis (Hemiptera, Corixidae) in South-West Spain
Source: PLoS One. 2013 May 17;8(5):e63105. doi: 10.1371/journal.pone.0063105 (PMC3656867; doi:10.1371/journal.pone.0063105)
Supplement: Table S2 — Significantly different mean CTmin (Estimated Marginal Means tests with Bonferroni correction) from Table 4 according to acclimation conductivity (C) and species (Sp: Trichocorixa verticalis verticalis or Sigara lateralis ). These tests refer to partial effects from the final model. (DOCX) [file pone.0063105.s004.docx]

**Table S2.**

| **conductivity vs species** | | | | | | |
| --- | --- | --- | --- | --- | --- | --- |
| C | (I)Sp | (J)Sp | Mean Difference  (I-J) | | Std. Error | Sig. |
| 1 | Sl | Tvv | -1.58 | | 0.43 | <0.001 |
| 4 | Sl | Tvv | -2.76 | | 0.43 | <0.001 |
| **species vs conductivity** | | | | | | |
| Sp. | (I)C | (J)C | Mean Difference  (I-J) | | Std.Error | Sig. |
| *Sl* | 4 | 18 | -1.49 | | 0.43 | 0.006 |
| *Tvv* | 12 | 4 | -1.59 | | 0.43 | 0.003 |
| **species** | | | | | | |
| (I)Sp. | (J)Sp. | Mean Difference (I-J) | | Std. Error | Sig. |  |
| *Sl* | *Tvv* | -1.24 | | 0.22 | <0.001 |  |
