# Supplementary material for: Does Ecophysiology Determine Invasion Success? A Comparison between the Invasive Boatman Trichocorixa verticalis verticalis and the Native Sigara lateralis (Hemiptera, Corixidae) in South-West Spain
Source: PLoS One. 2013 May 17;8(5):e63105. doi: 10.1371/journal.pone.0063105 (PMC3656867; doi:10.1371/journal.pone.0063105)
Supplement: Table S3 — Significantly different mean SCPs (Estimate Marginal Means tests with Bonferroni correction) from Table 5 according to acclimation temperature (T), acclimation conductivity (C) and species (Sp: Trichocorixa verticalis verticalis or Sigara lateralis ). These tests refer to partial effects from the final model. (DOCX) [file pone.0063105.s005.docx]

**Table S3.**

| **temperature vs species** | | | | | | |
| --- | --- | --- | --- | --- | --- | --- |
| T | (I)Sp. | (J)Sp. | Mean Difference (I-J) | | Std. Error | Sig. |
| 10 | Sl | Tvv | -3.59 | | 0.77 | <0.001 |
| 15 | Sl | Tvv | -3.98 | | 0.77 | <0.001 |
| 25 | Sl | Tvv | 2.37 | | 0.95 | 0.014 |
| **species vs temperature** | | | | | | |
| Sp | (I)T | (J)T | Mean Difference (I-J) | | Std. Error | Sig. |
| *Tvv* | 25 | 10 | -4.43 | | 0.95 | <0.001 |
|  |  | 15 | -5.35 | | 0.95 | <0.001 |
| **species vs conductivity** | | | | | | |
| Sp | (I)C | (J)C | Mean Difference (I-J) | | Std. Error | Sig. |
| *Tvv* | 18 | 1 | -3.38 | | 1.15 | 0.026 |
|  |  | 4 | -5.15 | | 1.15 | <0.001 |
|  |  | 12 | -4.37 | | 1.15 | 0.002 |
| **conductivity vs species** | | | | | | |
| C | (I)Sp | (J)Sp | Mean Difference (I-J) | | Std. Error | Sig. |
| 4 | *Sl* | *Tvv* | -4.31 | | 0.89 | <0.001 |
| 12 | *Sl* | *Tvv* | -3.51 | | 0.89 | <0.001 |
| **conductivity** | | | | | | |
| (I)C | (J)C | Mean Difference (I-J) | | Std. Error | Sig. |  |
| 4 | 18 | 2.00 | | 0.73 | 0.044 |  |
| **temperature** | | | | | | |
| (I)T | (J)T | Mean Difference (I-J) | | Std. Error | Sig. |  |
| 25 | 15 | -2.17 | | 0.61 | 0.002 |  |
| **species** | | | | | | |
| (I)Sp | (J)Sp | Mean Difference (I-J) | | Std. Error | Sig. |  |
| *Sl* | *Tvv* | -1.73 | | 0.48 | 0.001 |  |
